# Chemoradiation in patients with isolated recurrent pancreatic cancer - therapeutical efficacy and probability of re-resection

**DOI:** 10.1186/1748-717X-8-27

**Published:** 2013-01-31

**Authors:** Daniel Habermehl, Ingo C Brecht, Frank Bergmann, Thomas Welzel, Stefan Rieken, Jens Werner, Peter Schirmacher, Markus W Büchler, Jürgen Debus, Stephanie E Combs

**Affiliations:** 1 Department of Radiation Oncology, University Hospital of Heidelberg, Im Neuenheimer Feld 400, Heidelberg, 69120, Germany; 2 Institute of Pathology, University of Heidelberg, Im Neuenheimer Feld 220/221, Heidelberg, 69120, Germany; 3 Department of Visceral Surgery, University Hospital of Heidelberg, Im Neuenheimer Feld 110, Heidelberg, 69120, Germany

## Abstract

**Background:**

In the present retrospective analysis we analysed the therapeutic outcome of a set of patients, who were treated with chemoradiation (CRT) for recurrent pancreatic cancer (RPC) in a single institution.

**Patients and Methods:**

Forty-one patients had a history of primary resection for pancreatic cancer. In case of an unresectable recurrency patients were treated with CRT at our institution between 2002 and 2010 with a median dose of 48.4 Gy (range 39.6–54 Gy). Concurrent chemotherapy regimes included Gemcitabine (GEM) in 37/41 patients (90%) and Fluorouracil (FU) or Capecitabine (CAP) in 4/41 patients (10%). Patients were re-evaluated after CRT with computed tomography and/or explorative laparotomy. During re-resection or laparotomy 15 patients received an additional intraoperative radiotherapy (IORT) with a median dose of 15 Gy (range 12–15 Gy). Median age was 65 years (range 39–76 years) and there were 26 male and 15 female patients.

**Results:**

The median overall survival (mOS), local control (LC) and progression-free survival (PFS) were 16.1, 13.8 and 6.9 months respectively for all patients after the first day of CRT. Re-resection was possible in five patients (12%) and a complete remission (CR) as defined by tumor-free biopsy was seen in 6 patients (15%). When re-resection could be achieved after CRT mOS was improved to 28.3 months (n = 5 patients, 95%-CI 10.2 – 46.3 months). Patients receiving IORT had a significantly improved mOS compared to no IORT (p = 0.034). Fifteen patients (37%) experienced a local tumour progression and main site of distant metastasis was the liver (11 patients, 27%).Overall treatment-related toxicity was mild, grade III hematologic toxicity was observed in 11 patients (27%).

**Conclusion:**

In summary we observed a good therapeutic response with mild to moderate toxicity levels for CRT in RPC. Overall survival and PFS were clearly improved in case of induction of a complete remission (tumor-free biopsies) or after achieving a re-resection, thus providing a curative intended therapy even in case of disease recurrence.

## Introduction

Pancreatic cancer (PAC) is the fourth leading cause of cancer-related death in the Western World and still has, in spite of medical progress in the fields of diagnosis and treatment of this disease over the last decades, a poor prognosis [1]. Most patients lack early disease-related symptoms and present with advanced disease. At first diagnosis, only 10–20% of patients are eligible for curative resection, thus leading to an exceptionally grim 5-year survival rate of about 4–6% [2,3]. Even after curatively intended treatment of the primary tumor, local recurrence of the disease occurs in most cases (range 35–86%), [4,5].

Surgical approaches such as extended surgical resection or wider lymph node dissection were not successful with respect to a reduction of recurrent disease or a prolonged disease-free interval [6]. Therefore tumour recurrence develops in almost all patients, and treatment choices are often limited. To date, FOLFIRINOX is the chemotherapeutic agent of choice for patients with metastating disease [7]. In the adjuvant therapy of curatively-intended resected pancreatic cancer GEM monotherapy remains the standard of care [8]. In an important large multicenter randomized clinical trial chemotherapy with six cycles of gemcitabine (GEM) was compared to observation alone after R0- or R1-resections and lead to a significant improved disease-free survival period of 13.4 to 6.9 months [9]. Furthermore, the large multicentric ESPAC-3-trial compared GEM treatment with fluorouracil plus folinic acid and found no difference in DFS and OS, but treatment related toxicity was clearly reduced in the GEM group (97 vs. 52 serious adverse events, p > 0.001) [10].

The role of radiotherapy (RT) for recurrent pancreatic cancer (RPC) is not conclusively defined yet and may provide a treatment approach especially for patients who seem unresectable in loco-regional disease and without distant metastases. In cases limited for surgical intervention radiotherapeutic treatment approaches remain the most potent option in achieving local tumour control. Additionally, chemoradiation (CRT) may be applied as a neoadjuvant approach to enable secondary surgical resection with curative intention. Due to downsizing strategies, tumour minimization can be achieved with sparing of formerly affected tissue such as critical vessels and thus encompassing resectability [11]. Achieving secondary resectability ideally alters the treatment approach to curatively intended therapy.

In the present work we examined the clinical results of patients with RPC treated with with RT or CRT for RPC.

## Patients and methods

Fourty-one patients with RPC and without distant metastases were treated with CRT at the University Hospital Heidelberg between January 2002 and December 2010. The median age was 64 years (range 39–76 years) and there were 26 male and 15 female patients. Patients’ characteristics are shown in Table 1. Mean and median follow-up period was 18.6 and 11 months, respectively.

**Table 1:** Patients’ characteristics

**Patient characteristics**	
**number**	41
**Gender**	
Male (number)	26 (63%)
Female (number)	15 (37%)
**Age [y]**	
Median age (range)	64 (39–76)

Y years.

All patients who received CRT had a history of surgery and resection for their primary tumour. None of the analysed patients had a history of abdominal RT. Evaluation of the histopathological resection status, TNM status as well as AJCC staging and histological grading were performed according to the criteria recommended by the WHO (2010) [12]. Usually, resected specimen were prepared and diagnosed according to a previously reported protocol [13]: In 25 cases tumour-free margins were achieved (R0). In 11 cases microscopic tumour residues were observed while in two cases, macroscopic tumour remains were left at the resection site (R2). In two cases a clear histopathological classification was not possible, thus marking the residual as Rx. In one patient resection status was unknown. Histopathological grading showed a well-differentiated tumour (G1) in one case, in 25 cases tumours were classified as G2 and 15 patients were scored G3.

Recurrence was defined as locoregional re-manifestation (pancreatic compartment, area of resected primary tumour and regional lymph nodes) of pancreatic cancer. The analyzed patients had all a history of resection for (PAC) and a total of 35 patients received adjuvant chemotherapy containing fluorouracil/folinic acid (FU/FA), capecitabine (CAP) or GEM (treatment details listed in Table 2). For six patients there was no information available on previous adjuvant treatment in the primary setting. Median time from surgery in the primary setting until first day of radiotherapy in case of relapse was 16.9 months (average time 21 months, range 3.1 – 79.7 months). Concurrent percutaneous fractionated RT was applied with a median dose of 48.4 Gy (range 39.6-54 Gy) in median single fractions of 1.8 Gy.

**Table 2:** Treatment details

**Treatment details**	
**Previous resection**	41
**Localisation**	
Head	30 (73%)
Body	7 (17%)
Tail	4 (10%)
**Resection status during primary resection**	
R0	25
R1	11
R2	2
Rx	2
unknown	1
**Histopathological grading**	
G1	1
G2	25
G3	15
**Age at time of primary resection [y]**	
Median (range)	62 (37–75)
**Adjuvant therapy after primary resection**	
Chemotherapy	
unknown	35 (85%)
	6 (15%)
**Median duration until local recurrence after primary resection [months]**	
Median (range)	17.2 (3.1–79.7)
**Radiotherapy**	
Median dose (range)	49.4 Gy (39.6–54)
**Concomitant Chemotherapy**	
Overall	41 (100%)
Gemcitabine	37 (90%)
5-FU/Capecitabine	4 (10%)
**Intraoperative RT**	
**Number of patients**	15 (37%)
**Median dose (range)**	15 (12–15)
**Resection state after CRT**	
**total**	4
**R0**	3
**R1**	0
**R2**	2
**Rx**	0
**Negative biopsy during explorative laparoto my after CRT (number of patients)**	6 (15%)

CRT chemoradiation, Gy Gray, IORT intraoperative radiotherapy, RT radiotherapy.

Fifteen patients underwent at least an explorative laparotomy after CRT with the intention of re-resection. In six cases no residual tumor cells were seen after biopsy of suspected tumor masses, two patients underwent a R0-resection and in two further patients the residual tumor mass was reduced (partial resection, R2-resections). In one patient an involved lymph node was resected and intraoperative RT (IORT) was applied to the tumor bed. Five patients underwent IORT treatment in case of unresectability. Of the six patients with negative biopsies five patients received additional IORT during exploration. In the resected patients (2× R0, 2× R2) IORT was also performed. Additional IORT in case of re-resection or explorative laparotomy after CRT (not in the primary setting) was applied in 15 patients with a median dose of 15 Gy (range 12–15 Gy). Indication for IORT was decided by the surgeon and was usually performed when resection margins were expected to be positive for residual tumor tissue. However, this was left to the discretion of the treating surgeon and the individual patient and tumor characteristics during the operation. Surgery was performed whenever it was possible to achieve negative resection margins. Criteria for unresectability were in general the following: a more than 180 degrees encasement of the superior mesenteric artery, infiltration of the celiac trunk, an unreconstructible occlusion of the superior mesenteric vein or portal vein, aortic invasion or a surrounding tumour of parts of the abdominal aorta.

All patients received chemotherapy concomitantly. Concurrent chemotherapy was delivered in 37 cases as GEM with a dose of 300 mg/m^2^ weekly, followed by adjuvant cycles of full-dose GEM (1000 mg/m^2^) in 21 cases. Four patients received FU/FA or CAP. Additionally, 30 patients received full-dose GEM after completion of CRT, two patients received CAP and tow patients received further treatment with GEM plus Oxaliplatin or FOLFOX. Two patients received no further systemic treatment and for five patients details on further therapy was not available.

Local failure was characterized as size progression of the original recurrence mass, de-novo occurrence of a tumorous mass in the pancreatic compartment or infiltration in adjacent organs and in regional draining lymph nodes. Systemic failure was defined as metastatic progression in distant organs and as tumour cell dissemination into the peritoneum.

Overall survival and PFS were calculated from the first day of CRT until death or documented progression of disease. The log-rank test was performed to compare survival curves evaluating the association between clinical variables of interest and survival. All calculations were performed using the statistical software program SPSS 18.0 for Windows (Chicago, Illinois, US).

## Results

### Local control and progression-free survival

**Figure 1:**
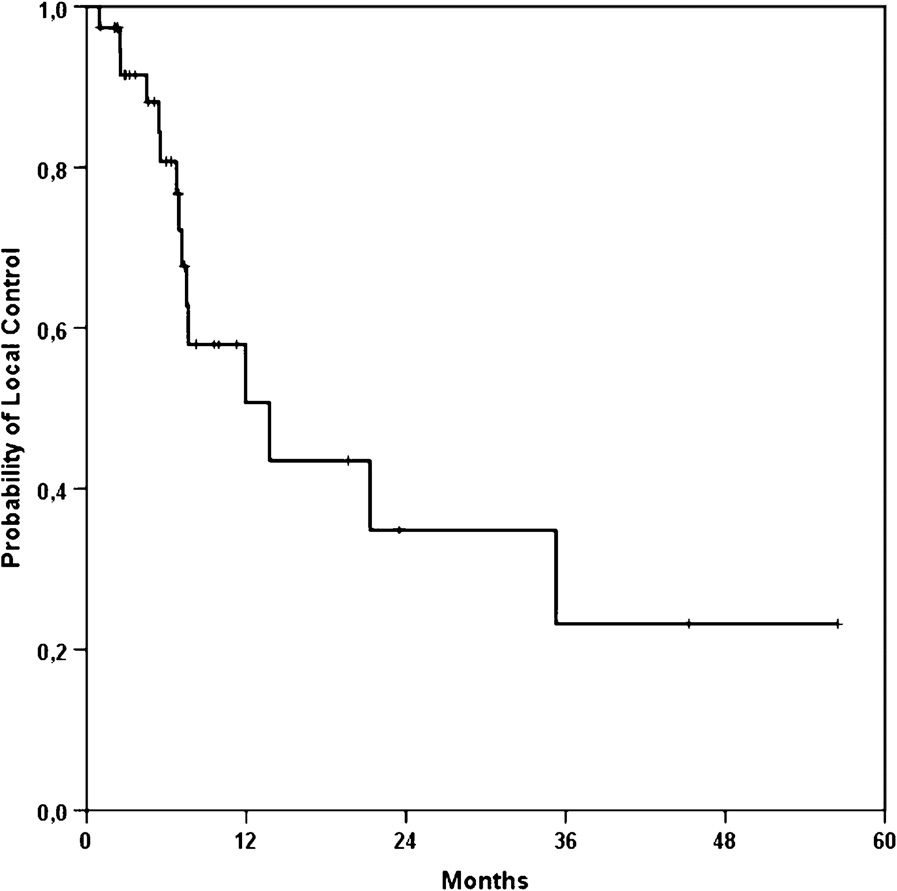
Kaplan-Meier curve for local control of all patients.

**Figure 2:**
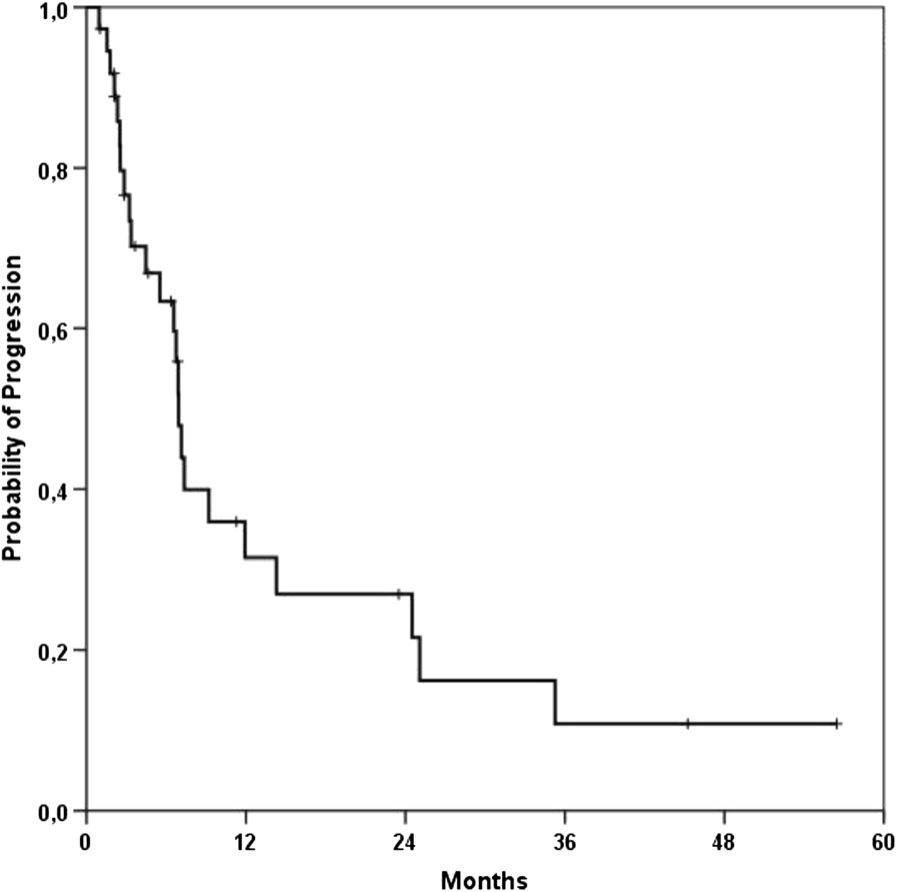
Kaplan-Meier curve for progression-free survival of all patients.

**Figure 3:**
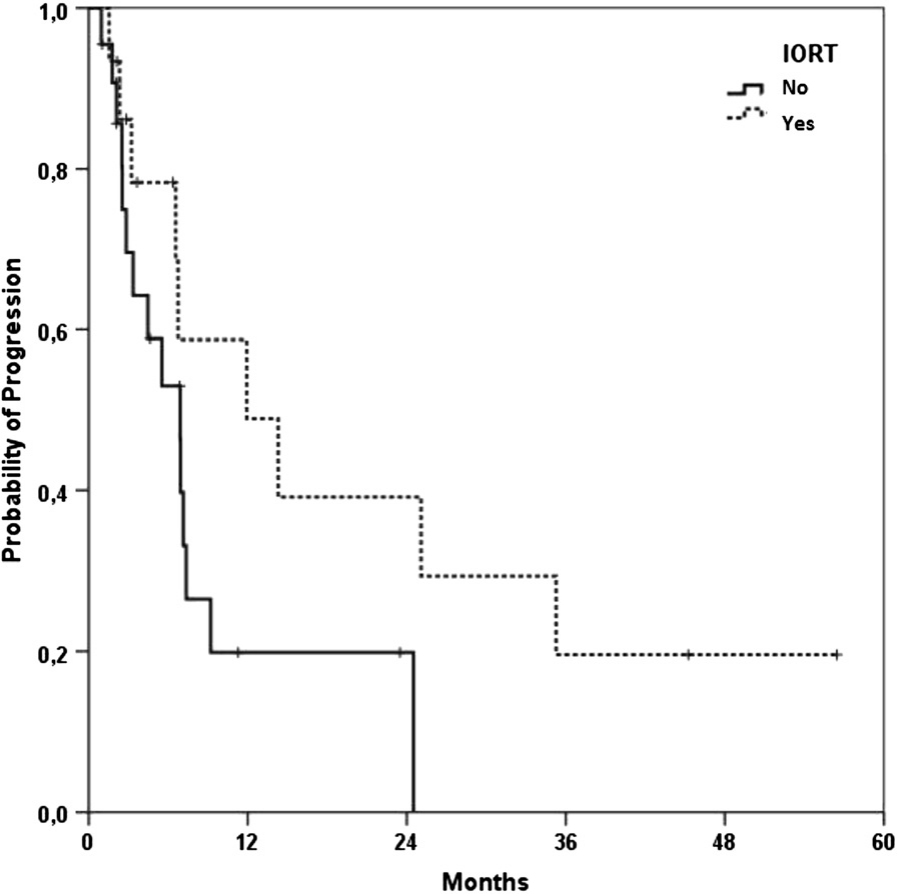
Kaplan-Meier curve for progression-free survival of all patients stratified byIORT treatment (yes vs. no).

Median local control for all patients was 13.8 months (95%-Confidence Interval 4.1 – 23.4 months; Figure 1). Progression-free survival was 6.9 months (95%-CI 6.3 – 7.5 months) (Figure 2). A re-resection was performed in five (12%) out of 41 patients and lead to a non-significant improvement of LC (21.3 vs. 13.8 months) and PFS (14.3 vs. 6.9 months); alas the follow-up time frame is still quite short for profounder observation. Fifteen patients underwent IORT after CRT in case of a resection or explorative laparotomy and lead to a clear but non-significant PFS improvement of 11.9 months compared to 6.9 months in the non-IORT group (p = 0.083) (Figure 3).

A total of 22 patients (54%) was diagnosed with distant failure over the course of their follow-up. Fifteen patients (37%) experienced a local tumour progression. Main site of disease progression was local in 15 (37%) patients while liver, peritoneal and pulmonal metastases were seen in 11 (27%), 6 (15%) and 5 (12%) patients, respectively.

### Survival

**Figure 4:**
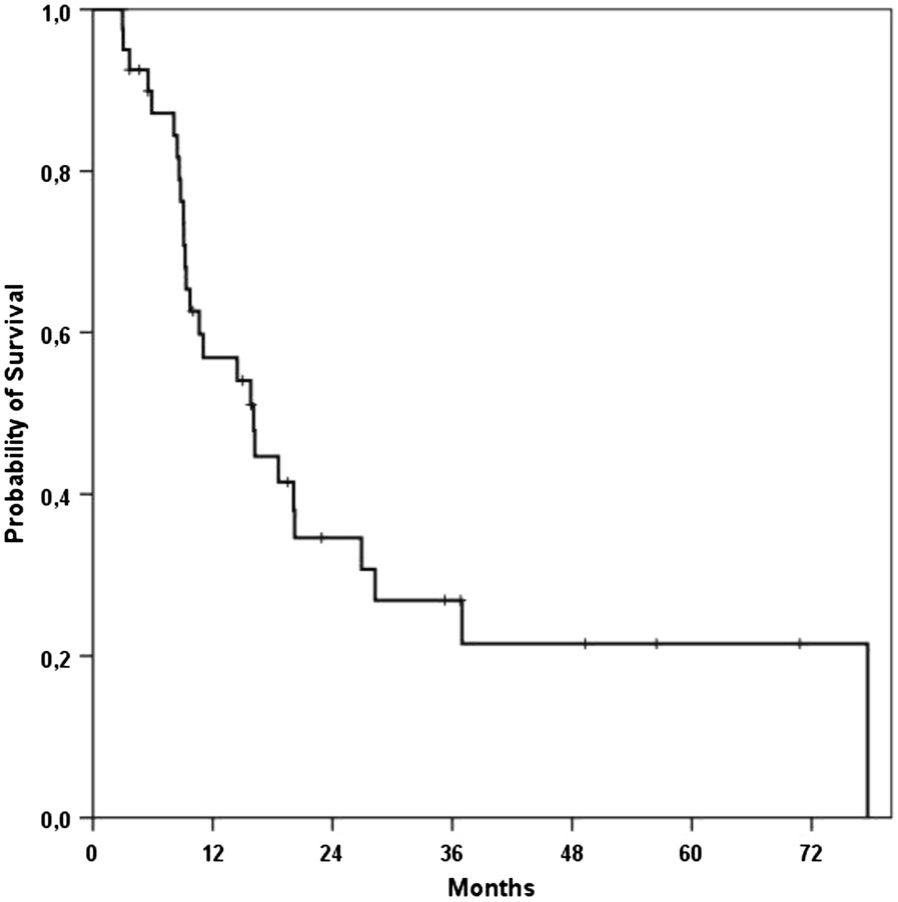
Kaplan-Meier curve for overall survival of all patients.

**Figure 5:**
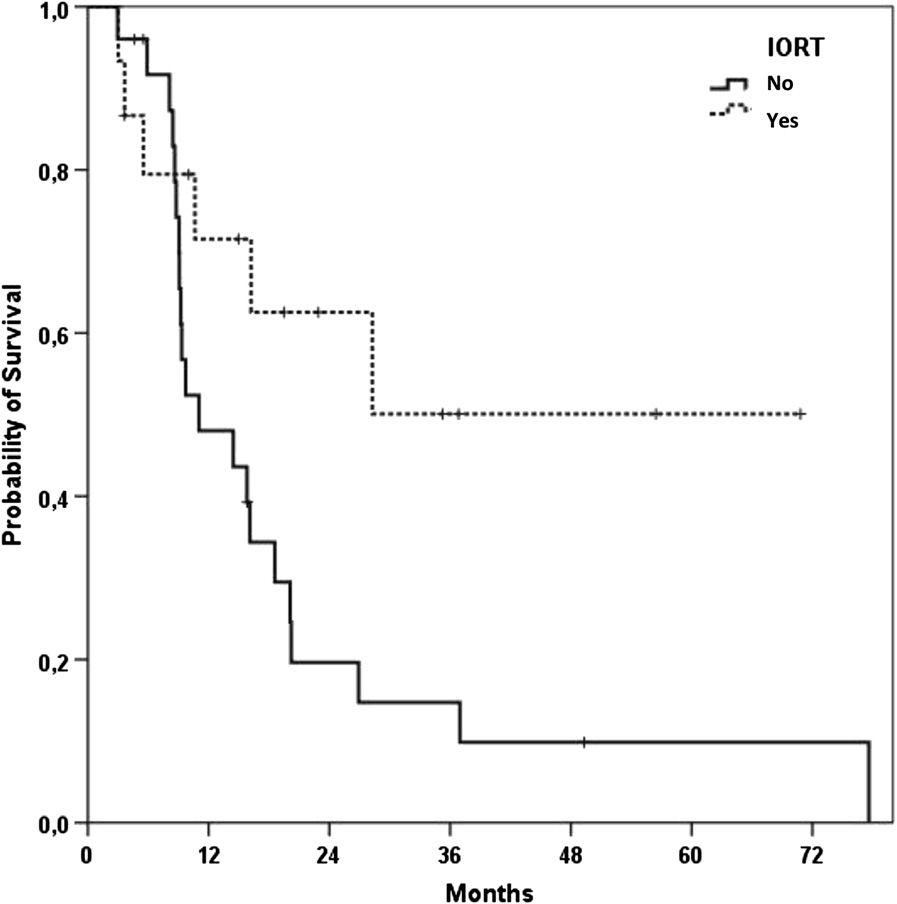
Kaplan-Meier curve for overall survival of all patients stratified by IORT treatment (yes vs. no).

Median overall survival (mOS) of all treated patients was 16.1 months (95%-CI, 9.2 – 23 months) after start of CRT (Figure 4). When re-resection could be achieved after CRT mOS was improved to 28.3 months (n = 5 patients, 95%-CI 10.2 – 46.3 months). Patients receiving IORT had a significantly improved mOS compared to no IORT (p = 0.034) (Figure 5). Furthermore, patients with any response to therapy as defined by re-resection, IORT treatment or absence of tumor tissue in a biopsy during explorative laparotomy had a sigificant improved mOS (p = 0.031).

### Toxicity

Side effects observed were mainly related to systemic treatment. Eleven patients had moderate hematologic toxicity (grade III), consisting of thrombocytopenia and neutropenia. Mild gastro-intestinal side effects (grade I-III) included nausea, vomiting, fatigue, diarrhoea and abdominal pain and was recorded in 22 patients (54%). Most frequent GI side effect was nausea in 17 patients (41%). In 11 (27%) patients, chemotherapy administration was reduced due to moderate hematologic or gastrointestinal toxicity. Seven patients had grade I (17%) and eight patients had grade II (20%) hematologic toxicity. In one patient, skin eruption became manifest during CRT. Eleven patients had grade III hematologic toxicity leading to a reduction or suspension of the concurrent chemotherapy in ten cases (27% of all patients treated with concomitant chemotherapy). The observed mild to moderate side effects could be treated successfully by supportive care.

## Discussion

In the treatment of RPC, there is by now no conclusively defined standard treatment protocol yet. Surgical resection with tumour free margins should be aimed in patients with primary diagnosed pancreatic cancer as well as in RPC patients but cannot be performed in all cases [14]. Our retrospective analysis of a comparable large cohort of patients shows the importance of achieving a (re-)resectability also in RPC patients leading to a remarkable median OS of 28.3 months and 16.7 months for all treated patients. Tumor-free biopsies could be achieved in 6 out of 41 patients (15%) during explorative laparotomy after CRT. Interestingly, IORT treatment lead to a significant improvement in OS (p = 0.035) but improvement of PFS was only seen by trends (p = 0.083).

In non-metastasized patients with unresectable RPC after primary curatively intended surgery, a locally intensified therapy can be justified. Therapeutic aim is therefore to provide a good LC which may lead to improved survival and PFS according to the treatment for primary locally advanced pancreatic carcinoma (LAPC) [11]. In a best-case scenario a complete remission or a secondary resectability could be achieved. In analogy to patients with newly diagnosed LAPC CRT protocols including GEM showed a clear survival advantage in case of re-resection which was also shown by data derived from our institution [11]. Especially in locoregionally limited recurrent disease without any distant metastases, the major treatment goal is the operative resection [15]. In the primary setting, Gillen et al. showed by a meta-analysis that especially patients with initially unresectable pancreatic carcinoma experienced a benefit from CRT [16]. One third of the patients treated with neoadjuvant CRT for their primary tumour gained secondary resectability after completion of their neoadjuvant therapy. Overall survival was then comparable to patients with a priori resectable tumours.

Wilkowski and colleagues examined outcome and toxicity of a comparable patient group with isolated local RPC that was treated with CRT protocols [17]. PFS and mOS were 14.7 and 17.5 months from start of CRT, respectively, and thus generally comparable to our findings. Main toxicity was hematological and consisted of grade III-IV leukopenia in 5 of 18 patients (28%, compared to 27% in our study) whereas no higher gastrointestinal side effects were seen (as in our study). Systemic disease progression was more pronounced than local progressive tumour growth (28.9% local relapse, 61.1% distant metastasis).

Only recently, an analysis of patients at the Heidelberg Medical Center treated with surgical interventions for recurrent pancreatic cancer could demonstrate promising results after resection in case of isolated RPC [18]. A high percentage of patients underwent re-resection (74%) successfully, while 16% could not undergo a surgical procedure. Median overall survival was significantly improved in resected patients (26 months vs. 10.8 months). In 15 of 103 patients a neoadjuvant CRT protocol was chosen in case of RPC and in 23 patients CRT was applied postoperatively. The present manuscript includes these surgically resected patients, all patients where an intervention was not possible, as well as an update of clinical data treated until December 2010 with longer follow-up; the analysis puts the focus on the radiooncological concept, toxicity and response.

A major problem in the treatment of pancreatic cancer is the high amount of recurrence even after curatively intended treatment with completely resected primary tumour. According to Sperti et al., up to 86% of patients treated with curative intention are at risk to develop a locoregional tumour recurrence [19]. Comparable results were referred by autopsy studies and showed a local or retroperitoneal tumour recurrence in 75–80% [5,20]. Other reports on patterns of failure after resection point to a predominant occurrence of distant metastasis, such as in the liver and peritoneum [4]. A recent meta-analysis on adjuvant treatment after primary resection compared CT and RT after curatively resected pancreatic carcinoma and shows a small advantage for CT only regarding PFS and OS [21]. In this context GEM has proven efficacy and an advantageous toxicity profile as single-agent adjuvant treatment as well as in combined CRT for LAPC [11,22]. Similar results concerning the good tolerability of GEM-based CRT were obtained by previous reports of our group and others [11,21,23].

The efficacy of an additional use of IORT remains an open question even if it is used as a common technique in RPC patients in several institutions. According to our analysis patients with RPC seem to benefit from IORT in terms of prolonged OS and PFS. This finding is in accordance with a recent multi-institutional review of 144 patients treated with IORT with our without EBRT in primary LAPC. Patients receiving IORT and EBRT as well as chemotherapy had a favourable OS and PFS [24]. However data on IORT treatment are mostly retrospective, patient cohorts are small, dose prescriptions are varying and target definition is hardly reproducible. Furthermore, application of IORT is limited to patients with local disease and can hardly be applied in case of locally advanced tumors. Nevertheless IORT is considered as a useful tool to gain local control with low toxicity rates in adjuvant and primary disease [24,25]. Finally, in our analysis patients that underwent IORT have comparable high rates of tumor-free biopsies (5 patients) and of re-resection (5 patients), thus representing a subgroup with a better response to therapy, in principle.

Our study was limited due to the retrospective design and the relatively low number of patients. Moreover there can be a substantial selection bias because only patients were selected for an intensified local therapy such as CRT which were in a comparatively good performance status and without distant metastases. However, all patients received chemotherapy concomitantly and were treated with a homogeneous treatment protocol consisting of combined CRT with GEM (37/41 patients) or FU/FA/CAP (4/41 patients). According to previous published data on primary LAPC from our institution this treatment approach is well tolerated and shows good response rates in pancreatic and hepatobiliary diseases [11,23].

In summary, our data provide a possible treatment opportunity in patients with locoregional recurrent pancreatic carcinoma with acceptable toxicity rates, good survival rates and the possibility of re-resection for initially unresectable patients. In accordance with other treatment opportunities, CRT can be successfully used in this setting.

## Competing interests

The authors declare that they have no competing interests.

## Authors’ contributions

DH, SR, TW, MWB, JW, FB, PS, JD and SEC were responsible for patient treatment and care. ICB and DH collected the patients’ data. DH performed all statistical analyses. DH and ICB wrote the manuscript. SR, TW, MWB, , JW, FB, PS, JD and SEC contributed to the analysis of data and revised the manuscript. SEC conceived the study, helped to write and finalized the manuscript. All authors helped with the interpretation of the data, read and approved the final manuscript.
